# Effects of Dietary Docosahexaenoic Acid (DHA) Levels on Growth Performance, Fatty Acid Profile, and NF-*κ*B/Nrf2 Pathway-Related Gene Expression of Razor Clam *Sinonovacula constricta*

**DOI:** 10.1155/2024/9107191

**Published:** 2024-10-28

**Authors:** Yuxiang Zhu, Kai Liao, Hailong Huang, Yang Liu, Yang Zhang, Deshui Chen, Bin Ma, Jilin Xu

**Affiliations:** ^1^School of Marine Sciences, Ningbo University, Ningbo 315211, China; ^2^Fujian Dalai Seed Science and Technology Co., Ltd, Ningde 352101, China; ^3^Laizhou Bay Marine Technology Co., Ltd, Yantai 261400, China

## Abstract

Dietary docosahexaenoic acid (DHA) is crucial for the optimal (Opt) growth of bivalves, but the precise dietary DHA requirement remains undetermined in bivalves. Our study identifies the optimal dietary DHA requirement for razor clam *Sinonovacula constricta* and demonstrates its effects on fatty acid profiles and gene expression related to inflammation and detoxification. Microencapsulated feeds with different DHA levels (DHA1–6 groups: 1.68, 4.85, 9.49, 12.6, 15.59, and 16.95 mg g^−1^ dry matter) were prepared using spray drying. Razor clams (initial wet weight: 3.8 ± 0.6 mg) were fed these microcapsules for a period of 20 days. The present study showed that the clams in the DHA1 group exhibited significantly lower weight and shell length gain rates compared to those in the DHA3, DHA4, DHA5, and DHA6 groups. Based on the shell length gain rate, the Opt dietary requirement of DHA for clam is approximately 6.42 mg g^−1^ dry matter. The clams in the DHA2 group had significantly higher crude lipid content compared to those in the DHA1 and DHA6 groups, while the clams in the DHA1 group had the highest ash content, significantly higher than that in the DHA4 and DHA6 groups. The DHA levels in the clams increased with the increase in DHA content in the microcapsules, while the levels of total *n*-6 polyunsaturated fatty acids (PUFAs), linoleic acid (LA), and alpha-linolenic acid (ALA) decreased. The mRNA levels of *cyclooxygenase-2* (*cox2*) and *5-lipoxygenase type 2 (5-lox-2)* were higher in the DHA1 and DHA6 groups compared to other microcapsule groups. As dietary DHA levels increased, the mRNA levels of *nuclear factor kappa B (nfκb*) and *nuclear factor erythroid 2-related factor 2 (nrf2*) decreased. Additionally, the mRNA levels of *glutamate-cysteine ligase* catalytic subunit (*gclc*) and *glutathione S-transferase* (*gst*) were highest in the DHA1 group. This is the first study to determine the Opt DHA requirement for juvenile razor clams using microcapsules with different DHA levels, and this study further reveals that dietary DHA can help reduce inflammation and oxidative status in clams.

## 1. Introduction

Docosahexaenoic acid (DHA) is a type of polyunsaturated fatty acids (PUFAs) crucial for the healthy growth of aquatic animals. Adequate intake of DHA enhances the activity and function of immune cells and strengthening the immune defence system [1]. Besides, it also possesses antioxidant and anti-inflammatory properties, mitigating the adverse effects of stress on aquatic animals and improving their survival rate and adaptability [2, 3]. However, excessive intake of DHA can lead to certain negative impacts, such as disrupt the balance of fatty acids, decreased growth rates, oxidative stress, and compromised immune function [4–7]. Meanwhile, there are studies indicating that increasing DHA beyond recommended levels is not harmful, but it also does not significantly improve growth performance of aquatic animals [8]. While research on invertebrate molluscs, especially bivalves, may be comparatively less extensive than that on fish, it is important to recognize that DHA is equally crucial for molluscs. As previous research has shown, when lacking DHA, the ash-free dry weight of *Crassostrea gigas* spat tended to be relatively low, while the mortality rate of mussel larvae *Mytilus galloprovincialis* significantly increases [9, 10]. Additionally, the lack of DHA in the feed led to the halt of growth of *Cerastoderma edule* [11]. Excessive DHA intake led to a decrease in the growth rate of abalones *Haliotis discus hannai*, with optimal (Opt) DHA requirements (% dry matter) being 1.32% for large-sized abalones and 0.88% for small-sized ones [6]. Microalgae with particularly high DHA content are often not the Opt feed for bivalve aquaculture [12, 13]. Therefore, the diet that maintains Opt DHA levels can not only prevent adverse effects such as inflammation and oxidative stress but also reduce costs.

Cyclooxygenase 2 (COX2) and lipoxygenases (LOXs) catalyse the production of eicosanoids from PUFAs, playing crucial roles in lipid metabolism, inflammation, and immune regulation [14–16]. The nuclear factor-kappa B (NF-*κ*B) pathway is a critical signalling cascade involved in regulating inflammation, modulating the transcription of pro-inflammatory cytokines such as COX2 [17]. The nuclear factor erythroid 2-related factor 2 (Nrf2) pathway is another crucial cellular signalling pathway primarily involved in regulating the cellular antioxidant response and activating phase II detoxification enzymes such as NAD(P)H: quinone oxidoreductase 1 (NQO1), glutamate-cysteine ligase catalytic subunit (GCLC), and glutathione S-transferase (GST) [18, 19]. Relevant research has primarily focused on mammals and fish, but some studies have demonstrated the presence of NF-*κ*B and Nrf2 pathways in bivalve species, indicating their significant roles in inflammation, immunity, and redox regulation [20, 21]. However, studies on the effects of DHA on these two pathways in bivalves are relatively limited. According to our knowledge, there is currently only one study indicating that DHA treatment alleviated the stress caused by diarrhetic shellfish toxins on the mussel *Perna viridis* through the Nrf2 pathway [22]. However, there was no study demonstrating the effect of dietary DHA on inflammation and the NF-*κ*B pathway in bivalve species. Therefore, further research is needed to fully understand the impact of dietary DHA on inflammation, oxidative stress, and associated signalling pathways in bivalve species.

Currently, bivalve aquaculture relies heavily on microalgae, which constitute 50% of nursery operating costs [23]. However, largescale cultivation of microalgae is susceptible to contamination by pathogens, potentially leading to significant economic losses for nurseries. Therefore, there is an urgent need for the development of artificial feeds. Microcapsules are considered the most promising alternative to microalgae as feed for bivalves [23–29]. Additionally, microcapsules can be utilized to precisely study the nutritional requirements of bivalves. Based on our previous research, the Opt carbohydrate/lipid ratio for the razor clam *Sinonovacula constricta* has been determined to be 33% carbohydrates and 11% lipids [29]. However, the requirements of other important nutrients, such as *n*-3 PUFAs, remain undetermined for this species. The clam *S. constricta* is one of China's important economic bivalve species, with a production exceeding 800,000 tons in 2023 [20]. According to previous research, dietary DHA is essential for the development of razor clam due to their limited ability to synthesize it internally [30]. Juvenile clams fed with diets rich in DHA from *Isochrysis galbana* or eicosapentaenoic acid (EPA) from *Chaetoceros calcitrans* consistently exhibited higher growth rates compared to those fed with diets rich in linoleic acid (LA) and alpha-linolenic acid (ALA) from *Chlorella* sp., highlighting the importance of dietary *n*-3 PUFAs [31]. In another study, razor clams fed with the microalgae *Thalassiosira weissflogii*, *T. pseudonana*, and an unidentified diatom, which contained 5.0%, 5.5%, and 6.7% DHA of total fatty acids, respectively, showed the highest shell length growth, indicating that DHA-rich microalgae have high food value for clams [13]. However, razor clams fed with *I. galbana*, which had the highest DHA content, did not show the best shell length growth, possibly due to its limitations in other essential fatty acids like EPA and arachidonic acid (ARA). It suggested that a diet solely rich in DHA might not be sufficient for the optimal growth of juveniles [13]. Existing studies on razor clam nutrition predominantly relies on microalgae. However, the nutritional composition of microalgae cannot be precisely controlled, which may make it difficult to accurately determine nutritional requirements. This study utilized spray drying to produce microcapsules with varying levels of DHA, aiming to accurately assess the DHA requirements of razor clams. Additionally, we sought to determine whether appropriate dietary DHA supplementation could alleviate inflammation and oxidative status in razor clam.

## 2. Materials and Methods

### 2.1. Animal Ethics Statement

All animal experiments were conducted according to the Guide for the Care and Use of Laboratory Animals formulated by the Ministry of Science and Technology of China. The study was approved by the Ningbo University Laboratory Animal Centre (affiliated with the Zhejiang Laboratory Animal Common Service Platform), license number SYXK (ZHE 2008 ± 0110).

### 2.2. The Preparation of Microcapsule Feeds

The highest DHA levels are found in genus *Isochrysis*, reaching approximately 20 mg g⁻^1^ dry matter [32]. Therefore, we designed microcapsules with DHA levels ranging from 0 to 20 mg g⁻^1^ dry matter to accurately study the DHA requirements. Based on previous study, the lipid level was designed to be maintained at 11% dry matter [29]. To maintain consistent lipid levels, the LA/ALA-rich and DHA-rich oils were adjusted. All oils were purchased from Shanxi Guancheng Biological Technology Co., Ltd (Shanxi, China). The fatty acid compositions of these oils are shown in Table S1.

Defatted fish meal, *Spirulina* spp. powder, kelp powder, zeolite, soybean lecithin, choline chloride, vitamin C, vitamin premix, mineral premix, and amylopectin were mixed in the appropriate proportions. Then, they underwent ultrafine grinding to achieve particle sizes below 5 µm (YQ50-1, Saishan, China). Subsequently, sodium starch octenyl succinate and casein were completely dissolved in distilled water within a temperature range of 70–90°C. The above two mixtures were combined and water was added to achieve a final solution with a solid content of 30% (W/W). The solution underwent emulsification using a high-shear emulsifying machine (FJ-200, Huxi, China). Finally, the emulsion was subjected to spray-drying (HF-5GL, Hefan, China) at an outlet temperature of 90°C and an inlet temperature of 120°C. The resulting microcapsules from spray drying were stored in a dry environment. The formulation of the microcapsules is detailed in Table 1, while Table 2 presents the fatty acid composition. The method employed for determining fatty acids in the feeds aligns with that used for razor clam, as elucidated in the section on razor clam fatty acid determination. The DHA levels in the microcapsules (DHA1-6) were as follows: 1.68, 4.85, 9.49, 12.6, 15.59, and 16.95 mg g⁻^1^ dry matter. DHA6 had lower DHA level than expected, possibly due to losses of DHA during the spray process.

### 2.3. Feeding and Sampling

White tanks with a capacity of 35 L were used for cultivation, with the bottom lined with a 2-cm layer of mud from tidal flats (sieved through an 80-mesh sieve and sterilized at high temperature). There were 21 tanks in total, with three tanks assigned to each treatment, resulting in three replicates (*n* = 3). Each tank was stocked with 2 g of juveniles in total weight. The razor clams had an initial average shell length of 3.6 ± 0.2 mm and an average weight of 3.8 ± 0.6 mg (standard error, *n* = 3). All razor clam juveniles were obtained from a seedling farm in Ninghai, Ningbo, Zhejiang, China. Aerator pumps were used for 24 h to provide sufficient and stable dissolved oxygen. After a 2-day acclimation period, feeding commenced with various diets, comprising six types of microcapsules with differing DHA levels (DHA1-6) and mixed microalgae powder (MMP; consisting of *I. galbana*, *Phaeodactylum tricornutum*, and *Platymonas helgolandica* in a 1:1:1 ratio; Table 1). These microalgae powders were purchased from Guotou Biotechnology Investment Co., Ltd (Beijing, China). Seawater was changed twice daily, at 8 a.m. and 8 p.m., with a volume of approximately 15 L per tank (keeping the total seawater volume at around 30 L). After each water change, microcapsule and MMP feed were immediately provided. The feeding amount during the culture period was maintained at 0.3–0.7 g per 30 L of seawater. The salinity of the cultivation water was maintained at 19–21 parts per thousand (ppt), and the temperature ranged from 17 to 22°C.

After 20 days of continuous feeding, the juvenile clams underwent a 1-day starvation period, followed by measurements of their mean wet weight and mean shell length. To obtain the mean wet weight, 0.1 g of clams was taken from each tank and counted (this process was repeated five times for accuracy). The mean wet weight of clams for each tank was then calculated. To determine the final mean shell length, each tank was sampled by placing 0.1 g of razor clams into a culture dish filled with seawater. Approximately 30 individuals were randomly selected from the sample, and their shell lengths were measured under a stereomicroscope. The average shell length of these clams was recorded as the mean shell length for that tank of razor clams.

A total of 100 mg of clams was accurately weighed and placed into 1.5-mL RNase-free centrifuge tubes for gene expression analysis, with 10 tubes taken from each tank. The remaining clams were placed into 50-mL centrifuge tubes for proximate composition and fatty acid profile analysis.

### 2.4. Survival Rate and Growth Performance

The survival rate (%), mean wet weight (mg), weight gain rate (µg day^−1^), shell length (mm), and shell length gain rate (µm day^−1^) of the razor clams fed different diets were statistically analysed or calculated using the following formulas:

  $$\begin{array}{c} \text{Weight gain rate }\left(µ{g\text{ day}}^{-1}\right)=\left(\text{final weight}-\text{initial weight}\right)/\text{days}. \end{array}$$

  $$\begin{array}{c} \text{Shell length gain rate }\left({µm\text{ day}}^{-1}\right)=\left(\text{final shell length}-\text{initial shell length}\right)/\text{days}. \end{array}$$

By analysing the beak-line analysis, we aimed to determine the relationship between the shell length gain rate (µm day^−1^) and dietary DHA levels (mg g^−1^ dry matter), in order to identify the Opt DHA level in this experiment.

### 2.5. Proximate Composition and Fatty Acids Analysis

The entire clam (including shell and soft tissue) was used to determine its proximate composition and fatty acid composition. The clams in the 50-mL centrifuge tubes were freeze-dried to remove moisture, followed by grinding into powder. To determine the crude protein content, 100 mg of freeze-dried powder was analysed using the Kjeldahl method [33]. For crude fat determination, 200 mg of freeze-dried powder was subjected to analysis using the chloroform–methanol method [34]. The ash content was determined by incinerating 200 mg of dry powder at 550°C for 5 h using a muffle furnace.

Total fatty acids from freeze-dried powder were extracted using the following method: A sample weighing 20 mg was placed into a 10 mL glass tube. Then, 1 mL of *n*-hexane and 1.5 mL of acetyl chloride (mixed in a ratio of methanol to acetyl chloride as 10 : 1) were added to the tube. The mixture was vigorously vortexed for 30 s to ensure thorough dissolution of the sample. Subsequently, the sample was heated in a water bath at 60°C for 2 h to facilitate the conversion of fatty acids into their methyl esters. After cooling, 2.5 mL of 6% K_2_CO_3_ solution was added to neutralize any remaining acidity, followed by another vortexing step. The sample was then centrifuged at 3000 g for 10 min to separate the organic phase. The supernatant containing the methyl esters was carefully transferred to a 2 mL vial through a filter membrane. Finally, the extracted sample was ready for chromatographic analysis to determine the fatty acid composition. The methylated samples were analysed using an Agilent 7890B/7000C gas chromatography system equipped with an EI detector. The specific operating conditions and parameters were as described in previous study [35]. The concentration of each fatty acid was calculated using MassHunter Qualitative Analysis (B.07.00).

### 2.6. Quantitative Real-Time PCR (qPCR) Analysis

The qPCR analysis was conducted following the method described in a previous study [36]. Total RNA was extracted using the TRIzol reagent (Invitrogen, USA), and cDNA was synthesized from RNA using the Accurate Biology (AG, China) reverse transcription kit. qPCR was conducted in a 20-µL reaction system containing 2 µL of cDNA, 0.5 µL of each primer, 10 µL of TB Green Premix Ex Taq II (Takara, Japan), and 7 µL of diethylpyrocarbonate water in a quantitative thermal cycler (Longgene, China). A two-step duplex fluorescence quantitative method was carried out as follows: an initial denaturation step at 95°C for 2 min, followed by 40 cycles of denaturation at 95°C for 5 s, and a combined annealing and extension step at 60°C for 10 s. The 2^−*ΔΔ*CT^ method was used to calculate the fold changes in gene expression at the mRNA level.  Table 3 provides the specific primers used for amplifying the reference gene (*β-actin*) and the target genes. Inflammation-related genes included *cox2, 5-lox-2, 5-lox-3, 5-lox-4, toll-like receptor 4 (tlr4), inhibitor of κB kinase α (ikkα), and nfkb*. Oxidation-related genes included*kelch-like ECH-associated protein 1* (*keap1*), *nrf2*, *gclc*, *gst*, and *nqo1*. The primer sequences were designed using NCBI Primer-BLAST (https://www.ncbi.nlm.nih.gov/tools/primer-blast/) with reference to the complete genome sequence of the clam *S. constricta*.

### 2.7. Statistical Analysis

The data analysis was conducted using the statistical package for the social sciences (SPSS) version 20. For each measured parameter, the mean and standard error were calculated (*n* = 3). The normality of the data was assessed using the Kolmogorov–Smirnov's test, while the homogeneity of variance was examined using Levene's test. In cases where the data did not follow a normal distribution and exhibited heterogeneity of variance, a log_10_ transformation was applied to meet the assumptions of normality and homogeneity of variance. Post hoc multiple comparisons were conducted using the Duncan's test, with a significance level set at *p* < 0.05. Significant differences between groups are indicated by different letters. Graphs were generated using GraphPad Prism (10.1.0).

## 3. Results

### 3.1. Survival and Growth Rate

**Figure 1:**
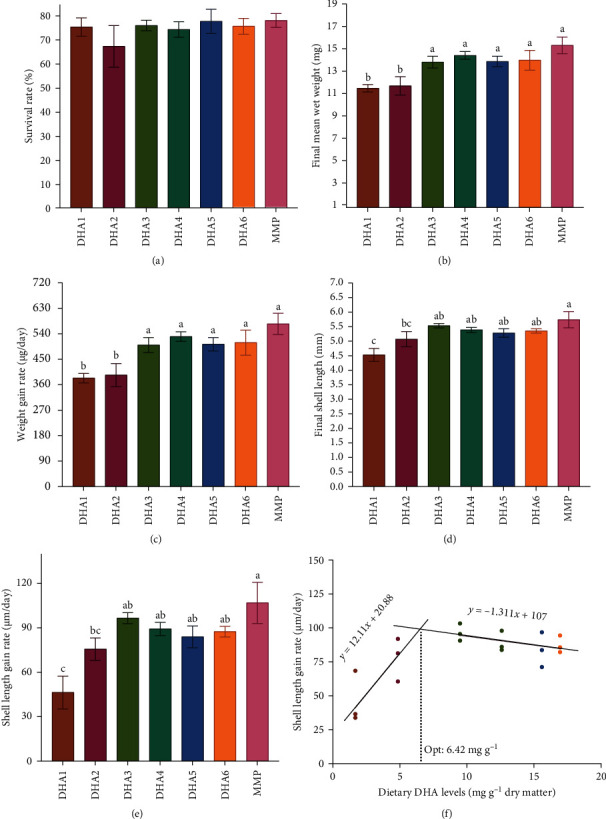
Effects of microcapsule feeds with different DHA levels (DHA1, DHA2, DHA3, DHA4, DHA5, and DHA6) and MMP diets on survival and growth performance of razor clam *S. constricta*. Bars are mean ± standard error, *n* = 3. Different letters show significant differences (*p* < 0.05). (A) survival rate (%); (B) final mean wet weight (mg); (C) weight gain rate (µg/day); (D) final shell length (mm); (E) shell length gain rate (µm/day); (F) relationship between dietary DHA levels and shell length gain rate. DHA, docosahexaenoic acid; MMP, mixed microalgae powder; Opt, the optimal level of DHA (mg g^−1^ dry matter).

The survival rates of juvenile clams showed no significant differences among all groups (*p* > 0.05, Figure 1A). The final wet weight and weight gain rate of the clams in the DHA1 and DHA2 group were significantly lower than those in the other groups (*p* < 0.05, Figure 1B, C). The final shell length and shell length gain rate of the clams in the DHA1 group were significantly lower than those in the other groups (*p* < 0.05), except for DHA2, where no difference was observed (*p* > 0.05, Figure 1D, E). A broken-line regression model based on the shell length gain rate showed that the optimum DHA content in microcapsule feeds for razor clam was 6.42 mg g^−1^ dry matter (Figure 1F).

### 3.2. Proximate Composition and Fatty Acid Profile

The crude protein content of clams in the MMP group was significantly higher than that in the other microcapsule groups (*p* < 0.05, Table 4). The crude lipid content of clams in the DHA2 group was significantly higher than that in the DHA1 and DHA6 groups (*p* < 0.05, Table 4). The ash content of clams in the DHA1 group was the highest, significantly higher than that in the DHA4, DHA6, and MMP groups (*p* < 0.05, Table 4).

When comparing the various microcapsule groups, the clams in the DHA1 and DHA2 groups contained the highest amount of LA (C18:2*n*-6), significantly higher than the other microcapsule groups (*p* < 0.05, Table 4). The clams in the DHA1 group contained the highest amount of ALA (C18:3*n*-3), significantly higher than the other microcapsule groups, while the lowest was found in the DHA6 group (*p* < 0.05, Table 4). The clams in the DHA5 and DHA6 groups contained the highest amount of DHA, significantly higher than the other microcapsule groups, while the lowest was found in the DHA1 group (*p* < 0.05, Table 4). Compared to clams fed with microcapsules, those fed with the MMP diet had a significantly higher content of tetradecanoic acid (C14:0), palmitoleic acid (C16:1), erucic acid (C22:1*n*-9), total monounsaturated fatty acids (MUFAs), ARA (C20:4*n*-6), and EPA (C20:5*n*-3) (*p* < 0.05, Table 4), and a significantly lower content of octadecanoic acid (C18:0), behenic acid (C22:0), oleic acid (C18:1*n*-9), LA (C18:2*n*-6), total *n*-6 PUFAs, and total PUFAs (*p* < 0.05, Table 4).

### 3.3. NF-*κ*B-Related Gene Expression

**Figure 2:**
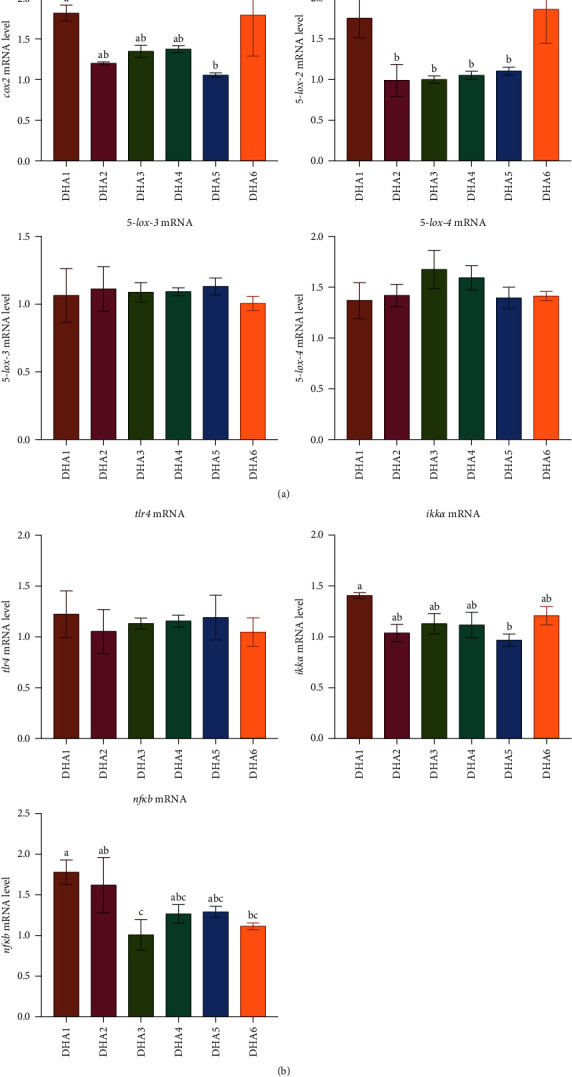
Effects of dietary DHA levels (DHA1, DHA2, DHA3, DHA4, DHA5, and DHA6) on mRNA levels of eicosanoid biosynthesis and NF-*κ*B pathway-related genes. Bars are mean ± standard error, *n* = 3. Different letters show significant differences (*p* < 0.05). (A) eicosanoid biosynthesis-related genes; (B) NF-*κ*B pathway-related genes. *5-lox-2, 5-lipoxygenase type 2; 5-lox-3, 5-lipoxygenase type 3; 5-lox-4, 5-lipoxygenase type 4; cox2, cyclooxygenase2;* DHA, docosahexaenoic acid; *ikkα, iκB kinase α subunit; nfκb, nuclear factor-kappa b; tlr4, toll-like receptor 4*.

The expression of the *cox2* in clams was significantly higher in the DHA1 and DHA6 groups compared to that in the DHA5 group, and the expression of the *5-lipoxygenase type 2* (*5-lox-2*) in clams was significantly higher in the DHA1 and DHA6 groups compared to that in the other microcapsule groups (*p* < 0.05, Figure 2A). The expressions of *5-lox-3* and *5-lox-4* in clams showed no significant differences among all microcapsule groups (*p* > 0.05, Figure 2A). The expression of the *tlr4* in clams showed no significant differences among all groups (*p* > 0.05, Figure 2B). The expression of the *ikkα* was significantly higher in the DHA1 group compared to the DHA5 group (*p* < 0.05, Figure 2B). As dietary DHA levels increased, there was a downward trend in the expression of the *nfκb*, and the clams in the DHA1 group showed significantly higher expression of *nfκb* compared to those in the DHA3 and DHA6 groups (*p* < 0.05, Figure 2B).

### 3.4. Nrf2-Related Gene Expression

**Figure 3:**
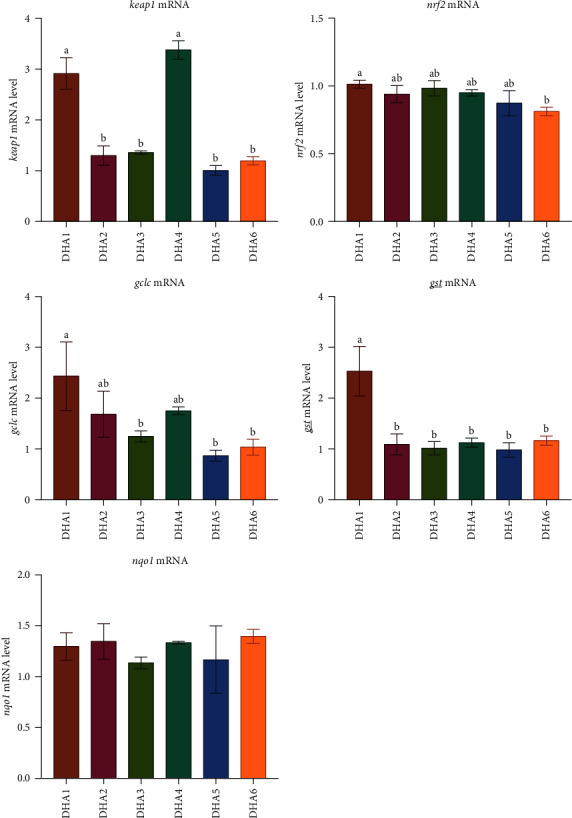
Effects of dietary DHA levels (DHA1, DHA2, DHA3, DHA4, DHA5, and DHA6) on mRNA levels of the Nrf2 pathway-related genes in razor clam *S. constricta*. DHA, docosahexaenoic acid; *gclc, glutamate-cysteine ligase catalytic subunit; gst, glutathione S-transferase; keap1, kelch-like ECH-associated protein 1; nqo1, NAD(P)H quinone dehydrogenase 1; nrf2, nuclear factor erythroid 2-related factor 2*.

The expression of the *keap1* in clams from the DHA1 and DHA4 groups was significantly higher compared to the other groups (*p* < 0.05, Figure 3). Clams from the DHA1 group showed significantly elevated expression of the *nrf2* compared to those from the DHA6 group (*p* < 0.05, Figure 3). The expression of the *gclc* exhibited a decreasing trend with increasing DHA levels in the feed, with significantly higher expression observed in the DHA1 group compared to the DHA3, DHA5, and DHA6 groups (*p* < 0.05, Figure 3). The expression of *gst* in the DHA1 group was significantly higher than in the other groups (*p* < 0.05, Figure 3). The expression of the *nqo1* in clams showed no significant differences among all groups (*p* > 0.05, Figure 3).

## 4. Discussion

### 4.1. Effects of Dietary DHA on the Growth and Fatty Acid Composition

Bivalves are renowned for their abundance of PUFAs, particularly DHA and EPA, both crucial for human nutrition [37]. The wealth of DHA and EPA in bivalves makes them highly valuable for human consumption, providing a natural and nutritious source of these essential fatty acids. These bivalve species obtain abundant PUFAs by feeding on phytoplankton, other planktonic organisms, and detritus [38]. Once ingested, PUFAs are efficiently absorbed and become crucial components of cell membranes. Previous research highlights the pivotal role of PUFAs content in microalgae diets for bivalve growth [39–41]. Microcapsules are considered the most promising alternative to microalgae as feed for bivalves. How to maintain the appropriate fatty acid composition, especially the PUFAs, is beneficial for its application. Meanwhile, microcapsules can be used to determine the nutritional requirements of specific nutrients in bivalves [29]. In this study, we utilized spray drying to prepare microcapsule feeds with varying levels of DHA to feed juvenile clams. Our research findings for the first time found that juvenile razor clams require approximately 6.42 mg g^−1^ dry matter (approximately 6% of the total fatty acids.). This is very similar to the DHA content of many diatoms, such as *T. weissflogii* (5.0% DHA of total fatty acids) and *T. pseudonana* (5.5% DHA of total fatty acids), which have been shown to have high food value for clams, leading to greater shell length gain rates [13]. Besides, this requirement of razor clam is lower than that of abalone *H. discus hannai*, which typically require 0.88%–1.32% of dry matter [6]. Juvenile razor clams need more total fat for growth compared to *H. discus hannai* [29]. Additionally, our results suggested that increasing DHA content did not significantly harm the growth of juvenile clams, consistent with some species such as sea bream *Sparus aurata* and fat snook*Centropomus parallelus* [8, 42]. This also explains why some microalgae, belonging to the family Isochrysidaceae and genus *Isochrysis*, with particularly high DHA content exhibit relatively good feeding efficacy, and compared to other nutritionally balanced microalgae diets, their effectiveness as clam feed does not significantly differ.

For aquatic animals, the fatty acid composition is determined by complex and dynamic interactions among various factors. Primary factors include dietary fatty acid intake, rates of oxidative catabolism of fatty acids, kinetics of desaturation and elongation reactions, as well as competitive incorporation and retro-conversions among fatty acids [5, 43]. In general, the fatty acid profile of aquatic animal mirrors that of the dietary sources. In the present study, some fatty acids in juvenile razor clams mirrored those in the microcapsule diets. Across microcapsule groups, there was a decreasing trend observed in levels of LA, total *n*-6 PUFAs, and ALA in clams, while DHA and total *n*-3 PUFAs exhibited an increasing trend. Additionally, significant differences were observed between the microcapsules and the MMP groups in terms of saturated fatty acids C14:0 and C18:0 levels. The C18:0 content was higher in individuals consuming microcapsule feeds, while those consuming microalgae exhibited higher C14:0 content. Those findings are consistent with the established observations by several authors that the fatty acid composition of tissue is directly influenced by the dietary fatty acid composition [44, 45]. However, some fatty acids such as C16:0, C22:1*n*-9, ALA, and ARA did not follow this trend when comparing the microcapsule groups with the MMP group. Despite the higher content of C22:1*n*-9, ARA, and ALA in the microcapsule feeds compared to the MMP, there were no higher levels of these fatty acids in the clams. Similar results were also observed in other clams fed different microalgae. For example, compared to the low ARA level diet (50% *C. gracilis* + 50% *Cyclotella nana*), the high ARA level diet (50% *Tisochrysis lutea* + 50% *C. gracilis*) resulted in a significantly lower ARA level in the hard clam, *Mercenaria mercenaria* [46]. We speculated that there may be differences in the metabolic pathways of C22:1n-9, ARA, and ALA between razor clams fed with microcapsules and those fed with microalgae in this study.

In the present study, the clams fed the low-DHA diet (DHA1) still maintained a DHA level of 3.6% of the total fatty acids. This suggests that razor clams have the ability to synthesize DHA on their own and highlights the importance of DHA for maintaining the clams' health and physiological status (as a certain level of DHA is necessary for their physiological activities). However, although razor clams can synthesize DHA, this capability was limited and insufficient to meet their developmental requirements, making dietary DHA necessary [30]. When we increased the DHA content in the microcapsules, there was a noticeable improvement in the clams' growth performance, and the relative DHA levels in the clams also increased. However, this increase in DHA levels was modest, and the extent of the increase was not substantial, indicating that excess DHA is likely to be metabolized. Previous study has shown that even when razor clams were fed *I. galbana*, which has an extremely high level of DHA, their body DHA levels were not significantly higher compared to those fed *C. calcitrans* [31]. All those findings suggested that the DHA content in *I. galbana* is excessive for razor clams, while our study provides a reference for the minimum DHA requirement for razor clams.

### 4.2. Effect of Dietary DHA on NF-*κ*B/Nrf2 Pathway-Related Gene Expression

Excessive inflammation poses health risks, regulated by the NF-*κ*B pathway, which orchestrates inflammatory responses [47, 48]. COX2 and LOXs are important regulatory enzymes of NF-*κ*B pathway [14, 49, 50]. We identified four isoforms of *lox* gene in the razor clam, but we were unable to categorize them distinctly, so we labelled them as *5-lox-1*, *5-lox-2*, *5-lox-3*, and *5-lox-4*. Previous studies have demonstrated that inflammation led to the upregulation of *nfκb* gene expression and *cyclooxygenase* activity in bivalve species [51, 52]. DHA has been demonstrated to possess anti-inflammatory effects and can inhibit the occurrence of various inflammatory reactions, including the suppression of the NF-*κ*B signaling pathway [3]. In the present study, we found that DHA had a certain inhibitory effect on the transcription levels of *nfκb* in the razor clam, reducing the transcription levels of *cox2*. This suggests that a diet rich in DHA could potentially reduce the inflammatory pathway in bivalves. Our finding is similar to previous research indicating that DHA inhibits NF-*κ*B pathway and reduces inflammatory responses in aquatic animals such as large yellow croaker *Larimichthys crocea*, Nile tilapia *Oreochromis niloticus*, and grass carp *Ctenopharyngodon idella* [44, 53, 54]. However, prior to this, it seems that there have not been any studies on the effects of dietary DHA on inflammation and NF-*κ*B pathway in bivalve species. Furthermore, our study is the first to investigate the expression of *loxs* in clam under different dietary fatty acid conditions. We found that the expression pattern of *5-lox-2* was consistent with that of *cox2*, suggesting its relevance to the inflammatory response in razor clams. To our knowledge, existing research has only demonstrated the presence of *lipoxygenase* in bivalves but has not explored the relationship between expression of *loxs* and dietary-induced inflammation in these species [55]. In summary, this study found that an appropriate level of dietary DHA can reduce the expression of inflammation-related genes such as *cox*, *5-lox-2*, and *nfκb*. Long-term inflammation could lead to metabolic disorders, affecting nutrient absorption and ultimately resulting in slower growth, which corresponds with our growth performance data. Additionally, inflammation often accompanies oxidative stress; thus, the upregulation of inflammation genes may increase the generation of free radicals, thereby raising the risk of cellular damage [56]. Clams might require additional antioxidant mechanisms to counteract this damage.

Nrf2 is also a crucial transcription factor primarily involved in regulating cellular oxidative stress responses [18]. Previous studies have demonstrated that the Nrf2 pathway in bivalves plays a role in antioxidant and detoxification defence systems. The expression of the *nrf2* in bivalves increases under toxic substance exposure [22, 57]. Although the variation in *nrf2* mRNA levels was not substantial in the present study, these mRNA levels did not fully represent the protein's functional activity [58]. Unfortunately, we do not have *S. constricta*-specific Nrf2 antibodies. GST, NQO1, and GCLC are downstream antioxidant components of Nrf2 pathway involved in phase II detoxification enzymes [59, 60]. In this study, mRNA levels of *gst* and *gclc* were elevated under low dietary DHA conditions. This indicates that under low dietary DHA conditions, razor clams are in a heightened oxidative state and experience oxidative stress, necessitating increased expression of *gclc* and *gst* to handle toxic substances such as free radicals and peroxide products. Previous results in the mussel *P. viridis* showed that DHA treatment might reduce oxidative stress and enhance detoxification by activating Nrf2 pathway and increasing the activity of downstream antioxidant enzymes [22]. This suggests that DHA treatment can increase transcription of the *nrf2* in bivalves. However, our results differ, possibly due to differences in experimental design. Our study did not involve short-term stress treatment; instead, long-term DHA feeding may have resulted in healthier clams, reducing the need for excessive activation of the Nrf2 pathway. Consequently, dietary DHA might not significantly upregulate Nrf2 pathway-related gene expression. Nonetheless, the antioxidant effects of DHA are consistent. Additionally, compared to fish fed palm or poultry oil, barramundi (*Lates calcarifer*) fed fish oil showed relatively lower GST activity [61]. Our study results indicate that increased DHA intake not only reduced inflammation levels but also lowered the oxidative-reduction state in razor clams.

## 5. Conclusions

This is the first study to determine that the Opt DHA requirement for juvenile razor clams is 6.42 mg g⁻^1^ dry matter. Additionally, the study revealed that dietary fatty acids significantly impact the fatty acid composition of razor clams, with DHA levels in clams increasing in response to higher dietary DHA content. Furthermore, appropriate DHA levels in the diet were found to reduce inflammation and oxidative stress in juvenile razor clams. These findings underscore the critical role of dietary DHA in enhancing clam health and highlight its potential to improve aquaculture practices by optimizing nutritional strategies for better growth.

## Figures and Tables

**Table 1:** Ingredients and proximate composition (% dry matter) of the microcapsule with different DHA levels (DHA1, DHA2, DHA3, DHA4, DHA5, and DHA6) and MMP diets.

Ingredients (%)	Diets						
	DHA1	DHA2	DHA3	DHA4	DHA5	DHA6	MMP
Defatted fish meal^a^	12.40	12.40	12.40	12.40	12.40	12.40	0.00
*Spirulina* spp. powder	5.00	5.00	5.00	5.00	5.00	5.00	0.00
Kelp powder	25.00	25.00	25.00	25.00	25.00	25.00	0.00
Zeolite	3.00	3.00	3.00	3.00	3.00	3.00	0.00
Soybean lecithin	0.50	0.50	0.50	0.50	0.50	0.50	0.00
Choline chloride	1.00	1.00	1.00	1.00	1.00	1.00	0.00
Vitamin C	1.50	1.50	1.50	1.50	1.50	1.50	0.00
Vitamin premix^b^	1.00	1.00	1.00	1.00	1.00	1.00	0.00
Mineral premix^c^	1.00	1.00	1.00	1.00	1.00	1.00	0.00
Amylopectin	5.10	5.10	5.10	5.10	5.10	5.10	0.00
SSOS	12.33	12.33	12.33	12.33	12.33	12.33	0.00
Casein	24.67	24.67	24.67	24.67	24.67	24.67	0.00
Soyabean oil	4.00	4.00	4.00	4.00	4.00	4.00	0.00
LA/ALA-rich oil	2.50	2.00	1.50	1.00	0.50	0.00	0.00
DHA-rich oil	0.00	0.50	1.00	1.50	2.00	2.50	0.00
EPA-rich oil	1.00	1.00	1.00	1.00	1.00	1.00	0.00
*Isochrysis galbana* powder	0.00	0.00	0.00	0.00	0.00	0.00	33.33
*Phaeodactylum tricornutum* powder	0.00	0.00	0.00	0.00	0.00	0.00	33.33
*Platymonas helgolandica* powder	0.00	0.00	0.00	0.00	0.00	0.00	33.33
Proximate composition (% dry matter)							
Crude protein^d^	36.60	36.07	36.13	36.20	36.53	35.83	43.40
Crude lipid^e^	10.81	11.34	11.73	11.44	11.36	10.60	17.10

Abbreviations: ALA, alpha-linolenic acid; DHA, docosahexaenoic acid; EPA, eicosapentaenoic acid; LA, linoleic acid; MMP, mixed microalgae powder; SSOS, starch sodium octenyl succinate.

^a^Defatted fish meal: 77.30% crude protein and 2.24% crude lipid.

^b^Vitamin premix (mg or g/kg): carotene 0.1 g, vitamin D 0.05 g, tocopherol 0.38 g, vitamin B1 0.06 g, vitamin B2 0.19 g, vitamin B6 0.05 g, cyanocobalamin 0.1 mg, biotin 0.01 g, inositol 3.85, niacin acid 0.77 g, pantothenic acid 0.27 g, folic acid 0.01 g, chloride choline 7.87 g, and cellulose 1.92 g.

^c^Mineral premix: (mg or g/kg): NaF 2 mg, KI 0.8 mg, CoCl_2_ · 6H_2_O (1%) 50 mg, CuSO_4_ · 5H_2_O 10 mg, FeSO_4_ · H_2_O 80 mg, ZnSO_4_ · H_2_O 50 mg, MnSO_4_ · H_2_O 60 mg, MgSO_4_ · 7H_2_O 1200 mg, Ca (H_2_PO_4_)_2_·H_2_O 3000 mg, NaCl 100 mg, and zeolite powder 15.447 g.

^d^Crude protein: Kjeldahl method.

^e^Crude lipid: Soxhlet extractor method.

**Table 2:** Fatty acid compositions (mg g^−1^ dry matter) of the microcapsule with different DHA levels (DHA1, DHA2, DHA3, DHA4, DHA5, and DHA6) and MMP diets.

Fatty acid compositions (mg g^−1^ dry matter)	Diets						
	DHA1	DHA2	DHA3	DHA4	DHA5	DHA6	MMP
C12:0	0.12 ± 0.01	0.22 ± 0.00	0.13 ± 0.01	0.21 ± 0.01	0.14 ± 0.01	0.14 ± 0.01	0.00 ± 0.00
C14:0	2.00 ± 0.02	2.03 ± 0.03	2.04 ± 0.04	1.94 ± 0.01	1.98 ± 0.01	1.78 ± 0.04	9.83 ± 0.05
C15:0	0.18 ± 0.01	0.18 ± 0.01	0.19 ± 0.01	0.18 ± 0.00	0.18 ± 0.00	0.16 ± 0.01	0.54 ± 0.03
C16:0	18.83 ± 0.07	21.06 ± 0.15	20.43 ± 0.00	19.28 ± 0.17	18.77 ± 0.08	17.57 ± 0.01	29.17 ± 0.03
C17:0	0.24 ± 0.01	0.25 ± 0.01	0.23 ± 0.00	0.23 ± 0.00	0.24 ± 0.01	0.20 ± 0.00	0.00 ± 0.00
C18:0	10.9 ± 0.14	12.44 ± 0.56	11.99 ± 0.22	11.15 ± 0.10	11.04 ± 0.05	10.43 ± 0.05	0.00 ± 0.00
C20:0	0.65 ± 0.02	0.68 ± 0.03	0.68 ± 0.05	0.65 ± 0.05	0.64 ± 0.00	0.60 ± 0.04	0.65 ± 0.05
C22:0	0.57 ± 0.02	0.58 ± 0.02	0.59 ± 0.00	0.56 ± 0.04	0.53 ± 0.04	0.50 ± 0.03	1.81 ± 0.08
Total SFAs	33.49 ± 0.22	37.43 ± 0.72	36.28 ± 0.24	34.21 ± 0.35	33.51 ± 0.16	31.39 ± 0.06	39.80 ± 0.59
C16:1	1.31 ± 0.04	1.34 ± 0.07	1.41 ± 0.03	1.43 ± 0.14	1.50 ± 0.01	1.38 ± 0.00	22.21 ± 1.16
C17:1	0.11 ± 0.00	0.11 ± 0.00	0.11 ± 0.03	0.09 ± 0.01	0.10 ± 0.02	0.10 ± 0.01	0.00 ± 0.00
C18:1*n*-9T	16.80 ± 0.03	17.75 ± 0.03	17.43 ± 0.52	16.73 ± 0.28	15.87 ± 0.25	14.81 ± 0.12	11.86 ± 0.13
C18:1*n*-9C	2.21 ± 0.02	2.14 ± 0.04	2.15 ± 0.10	2.20 ± 0.01	2.02 ± 0.02	1.80 ± 0.06	6.97 ± 0.08
C22:1*n*-9	0.57 ± 0.02	0.58 ± 0.07	0.59 ± 0.07	0.56 ± 0.03	0.53 ± 0.02	0.50 ± 0.03	0.10 ± 0.06
Total MUFAs	20.99 ± 0.12	21.92 ± 0.07	21.68 ± 0.28	21.00 ± 0.46	20.03 ± 0.30	18.58 ± 0.21	41.15 ± 1.05
C18:2*n*-6C	0.39 ± 0.04	0.31 ± 0.02	0.39 ± 0.05	0.41 ± 0.05	0.40 ± 0.06	0.37 ± 0.02	1.68 ± 0.13
C18:2*n*-6T (LA)	26.15 ± 0.52	27.52 ± 0.31	27.19 ± 0.49	25.64 ± 0.09	24.44 ± 0.00	22.71 ± 0.13	19.87 ± 0.25
C18:3*n*-6	1.18 ± 0.14	1.15 ± 0.05	1.16 ± 0.09	1.10 ± 0.09	1.05 ± 0.05	0.95 ± 0.00	0.00 ± 0.00
C20:4*n*-6 (ARA)	0.74 ± 0.09	0.64 ± 0.07	0.76 ± 0.06	0.80 ± 0.03	0.89 ± 0.07	0.86 ± 0.02	0.41 ± 0.07
Total *n*-6 PUFAs	28.45 ± 0.25	29.61 ± 0.18	29.5 ± 0.38	27.95 ± 0.02	26.76 ± 0.09	24.89 ± 0.14	22.29 ± 0.38
C18:3*n*-3 (ALA)	14.95 ± 0.04	12.54 ± 0.06	11.82 ± 0.60	9.86 ± 0.09	8.28 ± 0.16	5.26 ± 0.03	1.31 ± 0.04
C20:5*n*-3 (EPA)	8.98 ± 0.51	7.50 ± 0.58	9.00 ± 0.02	9.24 ± 0.03	9.88 ± 0.13	9.30 ± 0.27	31.45 ± 0.53
C22:6*n*-3 (DHA)	1.68 ± 0.22	4.85 ± 0.17	9.49 ± 0.03	12.60 ± 0.28	15.59 ± 0.58	16.95 ± 0.86	1.58 ± 0.05
Total *n*-3 PUFAs	25.61 ± 0.68	24.89 ± 0.81	30.31 ± 0.64	31.7 ± 0.17	33.75 ± 0.60	31.5 ± 0.62	41.76 ± 0.64
Total PUFAs	54.06 ± 0.43	54.50 ± 0.99	59.81 ± 1.02	59.65 ± 0.18	60.51 ± 0.69	56.39 ± 0.76	64.05 ± 0.26

*Note:* Each value represents the mean ± standard error, *n* = 3.

Abbreviations: ALA, alpha-linolenic acid; ARA, arachidonic acid; DHA, docosahexaenoic acid; EPA, eicosapentaenoic acid; LA, linoleic acid; MMP, mixed microalgae powder; MUFAs, monosaturated fatty acids; PUFAs, polyunsaturated fatty acids; SFAs, saturated fatty acids.

**Table 3:** Gene specific primers used for quantitative real-time PCR.

Target genes	Primer sequences (5′−3′)
*β-Actin*	F: CACTTCATGATGCTGTTGTATGTG
	R: GATTGTCAGAGACATCAAGGAGAAG

*cox2*	F: AAGCAACGCCGTCATGAAAC
	R: TCTGGTTTGAACTCCGTCCG

*nfκb*	F: GATACCTGATGGCGGTCCAG
	R: CAACCGCATACGGCTGATTG

*ikkα*	F: GTTCGATGCCTGGTTCAGGA
	R: AAGAGTGCCCACGAAGGATG

*tlr4*	F: ACCGGAAAACATTGCGTTCG
	R: GTCGCATTACCGTCACTGGA

*5-lox-2*	F: TCCAATATGGACGCCATCGG
	R: ACCACCGGCCATGTTGTATT

*5-lox-3*	F: GAACGGATGCCAACATTCGG
	R: TCACAATACCACCGACTGCC

*5-lox-4*	F: ACAAACATCGCACAGCTTGG
	R: CGGTACTTCGCCTCGGTATC

*keap1*	F: TTCACTCGCAAAGTCGGTGA
	R: ACACTGCGGAGATTCGTGTT

*nrf2*	F: GGCATCATAACTCCCTCCCC
	R: TGGAGAAGTGGGGACTGTCA

*nqo1*	F: GGTGTTCCCTCTGTACTGGC
	R: CTCCGAATACGCCCCTGTTT

*gclc*	F: CGTCTTCACGACAGCGGTAT
	R: TTCTACACGCCAGCCAATGT

*gst*	F: GTCGTCTTAACTGGGGTGGG
	R: GGGTATTGCAGACCTCCGAC

Abbreviations: *5-lox-2, 5-lipoxygenase type 2; 5-lox-3, 5-lipoxygenase type 3; 5-lox-4, 5-lipoxygenase type 4; cox2, cyclooxygenase 2; gclc, glutamate-cysteine ligase catalytic subunit; gst, glutathione S-transferase; ikkα, IκB kinase α subunit; nfκb, nuclear factor-kappa b; nqo1, NAD(P)H quinone dehydrogenase 1; nrf2, nuclear factor erythroid 2-related factor 2; tlr4, toll-like receptor 4*.

**Table 4:** Body compositions (% of dry weight) and fatty acid compositions (mg g^−1^ dry matter) of juvenile razor clam fed with microcapsules containing different levels of DHA (DHA1, DHA2, DHA3, DHA4, DHA5, and DHA6) and MMP diets.

Proximate composition (% of dry weight)	Diets						
	DHA1	DHA2	DHA3	DHA4	DHA5	DHA6	MMP
Crude protein	17.50 ± 1.26^bc^	19.17 ± 0.32^b^	18.30 ± 0.19^bc^	18.59 ± 0.63^bc^	16.99 ± 0.32^bc^	16.77 ± 0.19^c^	22.97 ± 0.63^a^
Crude lipid	6.26 ± 0.21^b^	7.85 ± 0.12^a^	6.61 ± 0.35^ab^	6.67 ± 0.65^ab^	7.00 ± 0.46^ab^	5.94 ± 0.34^b^	6.55 ± 0.17^ab^
Ash	57.50 ± 0.05^a^	52.87 ± 1.33^ab^	52.54 ± 1.88^ab^	49.76 ± 0.39^bc^	55.62 ± 0.60^a^	47.88 ± 0.89^bc^	44.73 ± 0.52^c^
Fatty acids (mg g^−1^ dry matter)							
C10:0	0.02 ± 0.01^a^	0.02 ± 0.00^a^	0.02 ± 0.00^a^	0.02 ± 0.01^a^	0.02 ± 0.01^a^	0.01 ± 0.00^a^	0.02 ± 0.01^a^
C11:0	0.01 ± 0.01^a^	0.00 ± 0.00^a^	0.00 ± 0.00^a^	0.00 ± 0.00^a^	0.00 ± 0.00^a^	0.00 ± 0.00^a^	0.00 ± 0.00^a^
C12:0	0.27 ± 0.01^a^	0.20 ± 0.00^c^	0.18 ± 0.01^d^	0.20 ± 0.01^c^	0.20 ± 0.00^c^	0.17 ± 0.01^d^	0.22 ± 0.01^b^
C13:0	0.02 ± 0.00^a^	0.02 ± 0.00^a^	0.02 ± 0.00^a^	0.02 ± 0.00^a^	0.02 ± 0.00^a^	0.02 ± 0.00^a^	0.03 ± 0.00^a^
C14:0	1.78 ± 0.01^c^	1.76 ± 0.01^c^	1.78 ± 0.23^c^	1.73 ± 0.11^c^	1.99 ± 0.08^b^	1.76 ± 0.05^c^	9.22 ± 0.04^a^
C15:0	0.63 ± 0.04^ab^	0.59 ± 0.02^b^	0.58 ± 0.08^ab^	0.58 ± 0.05^ab^	0.68 ± 0.05^a^	0.59 ± 0.09^ab^	0.62 ± 0.06^ab^
C16:0	13.27 ± 0.03^d^	15.00 ± 0.10^b^	14.50 ± 0.17^c^	15.36 ± 0.32^b^	16.34 ± 0.11^a^	14.27 ± 0.28^c^	14.1 ± 0.21^c^
C18:0	12.00 ± 0.04^c^	12.89 ± 0.03^b^	12.19 ± 0.16^c^	12.27 ± 0.15^c^	13.67 ± 0.23^a^	11.99 ± 0.02^c^	7.11 ± 0.13^d^
C20:0	0.27 ± 0.01^c^	0.35 ± 0.01^b^	0.34 ± 0.01^b^	0.37 ± 0.00^a^	0.38 ± 0.01^a^	0.33 ± 0.00^b^	0.28 ± 0.01^d^
C22:0	0.16 ± 0.01^c^	0.20 ± 0.00^b^	0.20 ± 0.01^b^	0.24 ± 0.01^a^	0.23 ± 0.00^a^	0.21 ± 0.01^b^	0.08 ± 0.01^d^
C24:0	0.10 ± 0.00^b^	0.10 ± 0.00^b^	0.10 ± 0.00^b^	0.10 ± 0.00^b^	0.10 ± 0.00^b^	0.11 ± 0.01^b^	0.17 ± 0.00^a^
Total SFAs	28.28 ± 0.15^d^	31.4 ± 0.04^b^	30.15 ± 0.05^c^	31.17 ± 0.45^b^	34.12 ± 0.42^a^	29.63 ± 0.24^d^	30.96 ± 0.04^b^
C16:1	0.62 ± 0.04^b^	0.47 ± 0.10^c^	0.51 ± 0.06^c^	0.51 ± 0.02^c^	0.57 ± 0.04^bc^	0.52 ± 0.01^c^	3.70 ± 0.07^a^
C18:1*n*-9T	2.44 ± 0.03^b^	2.81 ± 0.02^a^	2.45 ± 0.07^b^	2.52 ± 0.03^b^	2.31 ± 0.01^c^	1.88 ± 0.02^d^	0.93 ± 0.05^e^
C20:1	1.27 ± 0.01^b^	1.43 ± 0.56^ab^	1.45 ± 0.13^ab^	1.37 ± 0.08^ab^	1.56 ± 0.19^a^	1.28 ± 0.12^b^	1.23 ± 0.05^b^
C22:1*n*-9	0.06 ± 0.02^b^	0.06 ± 0.00^b^	0.06 ± 0.00^b^	0.06 ± 0.00^b^	0.06 ± 0.00^b^	0.07 ± 0.01^b^	0.20 ± 0.01^a^
Total MUFAs	4.40 ± 0.09^b^	4.78 ± 0.46^b^	4.40 ± 0.07^b^	4.50 ± 0.08^b^	4.52 ± 0.12^b^	3.74 ± 0.09^c^	6.06 ± 0.14^a^
C18:2*n*-6 (LA)	4.40 ± 0.31^a^	4.79 ± 0.28^a^	3.77 ± 0.30^b^	3.92 ± 0.10^b^	2.77 ± 0.28^c^	2.13 ± 0.18^d^	0.88 ± 0.13^e^
C18:3*n*-6	0.12 ± 0.01^b^	0.11 ± 0.01^b^	0.09 ± 0.01^c^	0.09 ± 0.01^c^	0.06 ± 0.01^d^	0.06 ± 0.00^d^	0.16 ± 0.01^a^
C20:2*n*-6	2.10 ± 0.17^b^	2.57 ± 0.05^a^	2.35 ± 0.01^b^	2.20 ± 0.10^b^	2.21 ± 0.13^b^	1.51 ± 0.02^c^	1.55 ± 0.09^c^
C20:3*n*-6	0.16 ± 0.01^b^	0.15 ± 0.00^b^	0.14 ± 0.01^bc^	0.12 ± 0.00^c^	0.13 ± 0.02^c^	0.13 ± 0.01^c^	0.21 ± 0.00^a^
C20:4*n*-6 (ARA)	1.70 ± 0.20^bc^	1.90 ± 0.01^b^	1.86 ± 0.07^b^	1.70 ± 0.03^c^	1.90 ± 0.11^b^	1.89 ± 0.04^b^	2.55 ± 0.04^a^
C22:2*n*-6	0.52 ± 0.02^a^	0.40 ± 0.01^b^	0.41 ± 0.06^b^	0.36 ± 0.04^b^	0.46 ± 0.03^b^	0.40 ± 0.04^b^	0.10 ± 0.00^c^
Total *n*-6 PUFAs	8.80 ± 0.32^b^	10.13 ± 0.21^a^	8.60 ± 0.36^b^	8.40 ± 0.10^b^	7.48 ± 0.14^c^	6.20 ± 0.18^d^	5.43 ± 0.10^e^
C18:3*n*-3 (ALA)	1.49 ± 0.02^a^	1.27 ± 0.01^b^	0.94 ± 0.06^c^	0.79 ± 0.02^d^	0.54 ± 0.02^e^	0.36 ± 0.03^f^	1.26 ± 0.04^b^
C20:3*n*-3	0.14 ± 0.01^c^	0.13 ± 0.00^c^	0.12 ± 0.01^cd^	0.10 ± 0.01^d^	0.10 ± 0.00^d^	1.77 ± 0.01^a^	0.21 ± 0.01^b^
C20:5*n*-3 (EPA)	3.40 ± 0.11^b^	3.42 ± 0.30^bc^	3.53 ± 0.04^b^	3.20 ± 0.48^bc^	3.40 ± 0.17^b^	3.10 ± 0.10^c^	4.33 ± 0.26^a^
C22:6*n*-3 (DHA)	3.60 ± 0.11^d^	4.91 ± 0.04^c^	5.20 ± 0.06^b^	5.31 ± 0.07^b^	5.89 ± 0.17^a^	5.70 ± 0.04^a^	3.67 ± 0.06^d^
Total *n*-3 PUFAs	8.63 ± 0.10^d^	9.72 ± 0.25^bc^	9.80 ± 0.02^bc^	9.40 ± 0.35^c^	9.88 ± 0.03^b^	10.44 ± 0.16^a^	9.50 ± 0.16^c^
*n*-3 *n*-6 PUFAs	0.98 ± 0.02^d^	0.96 ± 0.02^d^	1.14 ± 0.05^c^	1.12 ± 0.04^c^	1.32 ± 0.03^b^	1.69 ± 0.02^a^	1.76 ± 0.06^a^
Total PUFAs	17.42 ± 0.02^c^	19.85 ± 0.07^a^	18.34 ± 0.34^b^	17.51 ± 0.10^c^	17.40 ± 0.03^c^	16.62 ± 0.05^d^	15.00 ± 0.01^e^

*Note:* Values with different superscript letters within each row are statistically significant (one-way ANOVA, Duncan's test, *p*  < 0.05). Each value represents the mean ± standard error, *n* = 3.

Abbreviations: ALA, alpha-linolenic acid; ARA, arachidonic Acid; DHA, docosahexaenoic acid; EPA, eicosapentaenoic acid; LA, linoleic acid; MMP, mixed microalgae powder; MUFAs, monosaturated fatty acids; PUFAs, polyunsaturated fatty acids, SFAs saturated fatty acids.

## Data Availability

The data that support the findings of this study are available from the corresponding author upon reasonable request.

## Supporting Information

Supporting Information
*Table S1*: the fatty acid composition (%, total fatty acids) of different oils. All oils were purchased from Shanxi Guancheng Biological Technology Co., Ltd. The linoleic acid (LA)/alpha-linolenic acid (ALA)-rich oil was extracted from perilla seeds, while the eicosapentaenoic acid (EPA)-rich oil and docosahexaenoic acid (DHA)-rich oil were derived from microalgae.
