# Supplementary material for: Effects of Dietary Docosahexaenoic Acid (DHA) Levels on Growth Performance, Fatty Acid Profile, and NF-κB/Nrf2 Pathway-Related Gene Expression of Razor Clam Sinonovacula constricta
Source: Aquac Nutr. 2024 Oct 28;2024:9107191. doi: 10.1155/2024/9107191 (PMC11535280; doi:10.1155/2024/9107191)
Supplement: Supporting Information — Table S1: the fatty acid composition (%, total fatty acids) of different oils. All oils were purchased from Shanxi Guancheng Biological Technology Co., Ltd. The linoleic acid (LA)/alpha-linolenic acid (ALA)-rich oil was extracted from perilla seeds, while the eicosapentaenoic acid (EPA)-rich oil and docosahexaenoic acid (DHA)-rich oil were derived from microalgae. [file 9107191.f1.docx]

### Supplementary Table Caption

**SUPPLEMENTARY TABLE 1**: The fatty acid composition (% total fatty acids) of different oils. All oils were purchased from Shanxi Guancheng Biological Technology Co., Ltd. The linoleic acid (LA)/alpha-linolenic acid (ALA)-rich oil was extracted from perilla seeds, while the eicosapntemacnioc acid (EPA)-rich oil and docosahexaenoic acid (DHA)-rich oil were derived from microalgae.

**SUPPLEMENTARY TABLE 1**: The fatty acid composition (% total fatty acid) of different oils. All oils were purchased from Shanxi Guancheng Biological Technology Co., Ltd. The linoleic acid (LA)/alpha-linolenic acid (ALA)-rich oil was extracted from perilla seeds, while the eicosapentaenoic acid (EPA)-rich oil and docosahexaenoic acid (DHA)-rich oil were derived from microalgae.

| Fatty acid composition  (% total fatty acid) | Lipid substrates | | | |
| --- | --- | --- | --- | --- |
|  | EPA-rich oil | DHA-rich oil | LA/ALA-rich oil | Soybean oil |
| C11:0 | 0.00 | 0.00 | 0.00 | 0.00 |
| C12:0 | 0.00 | 0.00 | 0.00 | 0.00 |
| C14:0 | 0.34 | 1.61 | 0.24 | 0.30 |
| C15:0 | 0.03 | 0.08 | 0.03 | 0.08 |
| C16:0 | 1.91 | 4.08 | 2.81 | 14.36 |
| C17:0 | 0.24 | 0.12 | 0.10 | 0.44 |
| C18:0 | 3.75 | 1.22 | 1.25 | 11.72 |
| C19 | 0.00 | 0.00 | 0.00 | 0.00 |
| C20:0 | 0.62 | 0.20 | 0.12 | 1.44 |
| C21:0 | 0.06 | 0.04 | 0.01 | 0.14 |
| C22:0 | 0.13 | 0.06 | 0.00 | 1.63 |
| C23:0 | 0.00 | 0.00 | 0.00 | 0.20 |
| C24:0 | 0.00 | 0.00 | 0.00 | 0.56 |
| Total SFA | 7.09 | 7.40 | 4.56 | 30.86 |
| C16:1 | 0.70 | 3.11 | 0.96 | 0.31 |
| C17:1 | 0.04 | 0.03 | 0.11 | 0.22 |
| C18:1T | 6.60 | 4.13 | 11.78 | 24.28 |
| C18:1C | 2.62 | 1.47 | 0.00 | 0.35 |
| C22:1n-9 | 0.81 | 0.84 | 0.06 | 0.00 |
| Total MUFA | 10.77 | 9.58 | 12.90 | 25.16 |
| C18:2n-6T | 0.32 | 0.30 | 0.94 | 1.52 |
| C18:2n-6C (LA) | 1.20 | 0.63 | 23.56 | 31.64 |
| C20:3n-6 | 0.00 | 0.00 | 0.00 | 0.00 |
| C18:3n-6 | 0.18 | 0.16 | 1.47 | 2.07 |
| C20:4n-6 | 9.40 | 1.90 | 0.25 | 0.00 |
| C22:4n-6 | 0.17 | 0.83 | 0.02 | 0.00 |
| C22:5n-6 | 1.39 | 6.61 | 0.13 | 0.00 |
| Total n-6 PUFA | 12.66 | 10.43 | 26.37 | 35.23 |
| C18:3n-3 (ALA) | 0.00 | 0.00 | 49.39 | 8.75 |
| C18:4n-3 | 0.00 | 1.32 | 0.12 | 0.00 |
| C20:3n-3 | 0.56 | 0.11 | 0.24 | 0.00 |
| C20:4n-3 | 3.92 | 0.55 | 0.09 | 0.00 |
| C20:5n-3 | 49.48 | 11.31 | 2.47 | 0.00 |
| C22:5n-3 (EPA) | 2.63 | 9.18 | 0.48 | 0.00 |
| C22:6n-3 (DHA) | 12.88 | 50.13 | 3.38 | 0.00 |
| Total n-3 PUFA | 69.48 | 72.59 | 56.17 | 8.75 |
| Total PUFA | 82.14 | 83.02 | 82.54 | 43.98 |
